# Widespread Gene Conversion in Centromere Cores

**DOI:** 10.1371/journal.pbio.1000327

**Published:** 2010-03-09

**Authors:** Jinghua Shi, Sarah E. Wolf, John M. Burke, Gernot G. Presting, Jeffrey Ross-Ibarra, R. Kelly Dawe

**Affiliations:** 1 Department of Plant Biology, University of Georgia, Athens, Georgia, United States of America; 2 Department of Genetics, University of Georgia, Athens, Georgia, United States of America; 3 Molecular Biosciences and Bioengineering, University of Hawaii, Honolulu, Hawaii, United States of America; 4 Department of Plant Sciences, University of California, Davis, California, United States of America; Fred Hutchinson Cancer Research Center, United States of America

## Abstract

Centromeres are the most dynamic regions of the genome, yet they are typified by little or no crossing over, making it difficult to explain the origin of this diversity. To address this question, we developed a novel CENH3 ChIP display method that maps kinetochore footprints over transposon-rich areas of centromere cores. A high level of polymorphism made it possible to map a total of 238 within-centromere markers using maize recombinant inbred lines. Over half of the markers were shown to interact directly with kinetochores (CENH3) by chromatin immunoprecipitation. Although classical crossing over is fully suppressed across CENH3 domains, two gene conversion events (i.e., non-crossover marker exchanges) were identified in a mapping population. A population genetic analysis of 53 diverse inbreds suggests that historical gene conversion is widespread in maize centromeres, occurring at a rate >1×10^−5^/marker/generation. We conclude that gene conversion accelerates centromere evolution by facilitating sequence exchange among chromosomes.

## Abstract 2

Data from maize show that centromeres strongly suppress crossing over and instead undergo frequent genetic exchange in the form of gene conversion.

## Author Summary

Centromeres, which harbor the attachment points for microtubules during cell division, are characterized by repetitive DNA, paucity of genes, and almost complete suppression of crossing over. The repetitive DNA within centromeres appears to evolve much faster than would be expected for genetically inert regions, however. Current explanations for this rapid evolution tend to be theoretical. On the one hand there are arguments that subtle forms of selection on selfish repeat sequences can explain the rapid rate of change, while on the other hand it seems plausible that some form of accelerated neutral evolution is occurring. Here, we address this question in maize, which is known for its excellent genetic mapping resources. We first developed a method for identifying hundreds of single copy markers in centromeres and confirmed that they lie within functional domains by using a chromatin immunoprecipitation assay for kinetochore protein CENH3. All markers were mapped in relation to each other. The data show that, whereas classical crossing over is suppressed, there is extensive genetic exchange in the form of gene conversion (by which short segments of one chromosome are copied onto the other). These results were confirmed by demonstrating that similar short exchange tracts are common among the centromeres from multiple diverse inbred lines of maize. Our study suggests that centromere diversity can be at least partially attributed to a high rate of previously “hidden” genetic exchange within the core kinetochore domains.

## Introduction

In spite of their highly conserved function as the site of kinetochore assembly and spindle attachment, centromeres are the most dynamic regions of complex genomes. The components, copy number, and structural organization of centromeric DNA are highly divergent even among closely related species [1],[2],[3]. This apparent conflict between essentiality and sequence dispensability remains one of the major unresolved paradoxes in genetics. It has been hypothesized that the rapid evolution of centromeric DNA is primarily the result of an arms race in which meiotic drive sweeps novel centromeric repeats to fixation while centromeric proteins adapt to suppress this behavior [4]. Alternatively, some authors have argued that the role of selection is minimal and that observed variation can be explained by stochastic events such as mutation and genetic exchange [5],[6],[7]. Both proposals lack strong empirical support, as centromere drive has only rarely been documented [8], and mutational events are difficult to document in complex repetitive areas.

Centromeres are specified epigenetically by the presence of a centromere-specific histone H3 variant, CENH3, which organizes the overlying kinetochores [4]. Kinetochores affect the function and behavior of centromeric DNA in pronounced ways. Perhaps most notable is their effect on crossing over. Cytogeneticists have long known that centromeres severely repress meiotic crossing over [9], and this result has since been confirmed in all species studied [10],[11],[12]. As a consequence, centromeres are often defined as regions where the frequency of crossovers approaches zero [12],[13],[14]. Nevertheless it is not accurate to presume that centromeres never experience genetic exchange. Empirical studies have revealed evidence for recombination between sister centromeres [15],[16], gene conversion events have been inferred from sequence analysis of mammalian centromeres [17],[18],[19], and large intrachromosomal rearrangements have been documented in rice centromeres [20],[21]. However, despite the extensive circumstantial evidence for genetic exchange among centromeres, the frequency and nature of the recombination has been difficult to measure.

Maize centromeres contain a 156 bp tandem repeat known as CentC and an abundant class of *Ty3/Gypsy*-like transposons [22]. Several subfamilies of these so-called Centromeric Retroelements (CR elements, known as CRM in maize; [23]) exist, with CRM2 being the most abundant in the maize genome [24]. Over time, CR elements insert in and around each other resulting in a nested arrangement [25],[26]. Such insertion sites have a high probability of being unique and are generally polymorphic among lines, thereby providing an excellent tool for the genetic analysis of centromeres [27],[28]. Here we used transposon display [29] of CRM2 to generate centromere-specific markers in maize. Analysis of segregation in a mapping population, combined with CENH3 ChIP, allowed us to map the functional region of each maize centromere and provide direct evidence for conversion-type genetic exchanges within centromere cores. An analysis of haplotype variation and linkage disequilibrium in a broad panel of maize lines revealed further evidence for a high rate of gene conversion across all centromeres studied, consistent with an important role for stochastic processes in centromere evolution.

## Results

### Generating Unique Centromeric Markers Using CRM2-Display

Maize centromeres contain hundreds of retrotransposons of the CRM family, with clearly orthologous subfamilies present in rice [30]. Elements of the CRM2 subfamily account for a large proportion of these and exhibit very low transposition rates as judged by the small proportion of elements with insertion times in the past 75,000 years [30]. CRM2 thus has the features of an excellent genetic marker, being conserved enough to easily identify while still providing substantial polymorphism. Transposon display (known as TD; see [29]) makes it possible to capture such transposon-induced polymorphisms. By pairing a transposon-specific primer with a restriction site adapter, presence or absence of a particular insertion can be scored by resolving PCR products on a polyacrylamide gel. When we used TD to display all the CRM2 elements in the maize, we found that the number of products exceeded the resolution of our gel assays. To make the results manageable, we therefore added three selective bases to the adapter primer such that only 1/64 of the total number of bands was amplified in any given experiment. The resulting data suggest that 80.3% of the CRM2 bands are polymorphic between B73 and Mo17 (74 of 376 observed bands did not segregate).

**Figure 1:**
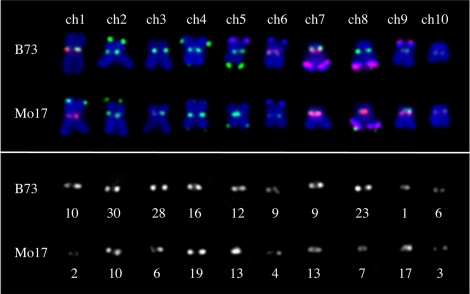
Correspondence between CRM2 marker number and CRM2 FISH intensity. Metaphase chromosomes from a B73/Mo17 hybrid line (from a single cell). CRM2 LTR and telomeres are shown in green, CentC and the knob 180 bp repeat are shown in red, and chromosomes are shown in blue. The lower panel shows CRM2 FISH signal (in white), and beneath each centromeric region is the total number of CRM2 TD markers recovered from that centromere.

To map CRM2 polymorphisms within centromeric regions, we scored a total of 257 CRM2 markers in 93 recombinant inbred lines from the maize IBM mapping population [31]. Of these, 238 mapped to 10 positions, each corresponding to a different maize centromere. The remaining 19 mapped at least one centimorgan outside of a centromere cluster and were classified as pericentromeric. The final data set revealed that the distribution of CRM2 markers is non-uniform among centromeres: there are 30 independent CRM2 markers on B73 centromere 2, for example, but only one marker on centromere 9. This result might be expected, as prior evidence has suggested repeat variation among maize centromeres [32]. An analysis of a B73/Mo17 hybrid line by fluorescent in situ hybridization (FISH) supports the interpretation that there is a rough correspondence between the number of markers recovered by CRM2 display and the intensity of CRM2 hybridization signal (Figure 1).

Recombinant inbred lines should be homozygous for markers from only one parent at the vast majority of loci. However, we also detected lines that contained markers characteristic of both (27 centromeres) or neither of the parental centromeres (6 centromeres). The former could be the result of residual heterozygosity, whereas the latter was presumed to represent contamination during the propagation of the lines. A combination of flanking centromeric markers and FISH (Figure S1) allowed us to confirm these expectations and remove the heterozygous and/or contaminant centromeres from consideration (Table S1). Overall centromeric heterozygosity was 2.15%, in line with expectations (2.5%) from a 6× self-crossed population.

### CRM2 Markers Interact with CENH3

**Figure 2:**
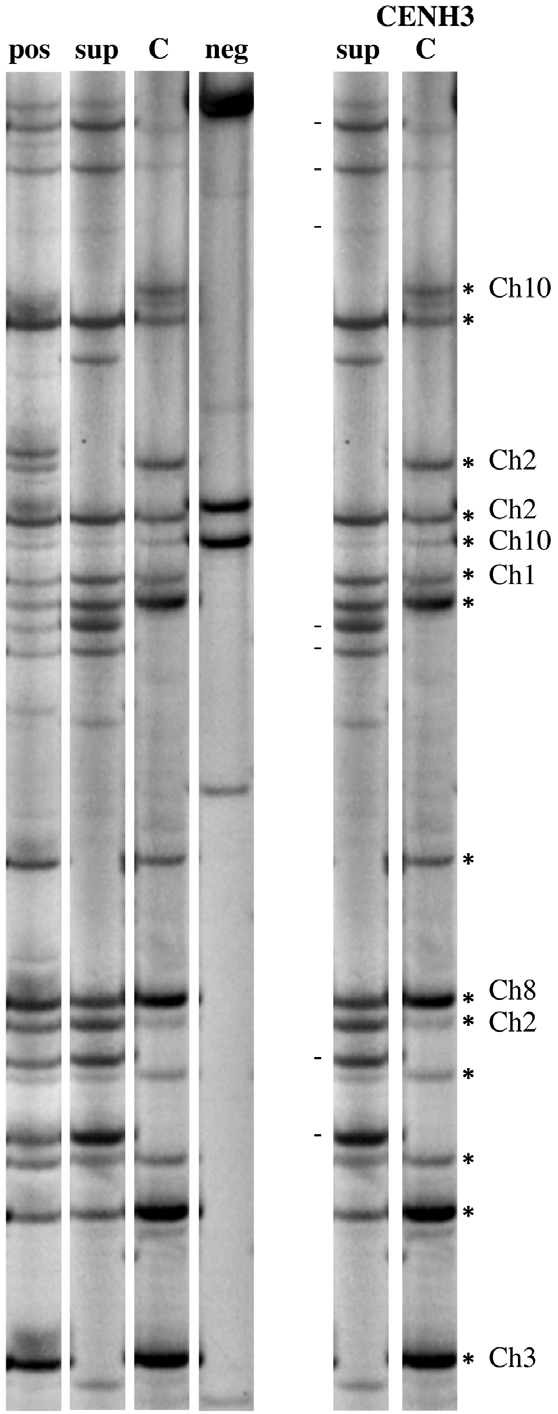
ChIP display. The image shows CRM2 elements labeled with P^33^ on a polyacrylamide gel. The left panel shows results from chromatin immunoprecipitation with controls: pos, B73 nuclei used for the ChIP experiment; sup, supernatant that did not bind to CENH3 antibodies; C, CENH3-bound markers; neg, no antibody control (shows non-specific binding to the sepharose beads used for precipitation). The right panel shows an annotated comparison between sup and C lanes. The chromosomal locations of the bands precipitated are indicated. The dashes next to the S lane denote non-precipitated bands.

CENH3 chromatin is not continuously distributed over centromeric domains, and any assay of common centromere repeats will thus provide only a partial view of the functional centromere/kinetochore regions. To identify CRM2 markers that lie within functional regions, we added a chromatin immunoprecipitation (ChIP) step to the protocol (Figure 2). Centromeric chromatin was precipitated with anti-CENH3 antibodies, the DNA purified from its associated chromatin, and the sample further processed for CRM2 display. Of 212 markers scored by ChIP, 122 were precipitated with CENH3 (57.5%), 40 were not precipitated with CENH3, and 50 gave inconsistent results among replicates. As expected, none of the 19 known pericentromeric bands was immunoprecipitated by CENH3 antibodies. These results are consistent with prior work showing that roughly 30% of maize CRM sequences can be immunoprecipitated by CENH3 antisera [23] and that a visible proportion of the CRM elements in maize are not associated with CENH3 [33].

### Sequence Conversion Events within Centromeres

**Figure 3:**
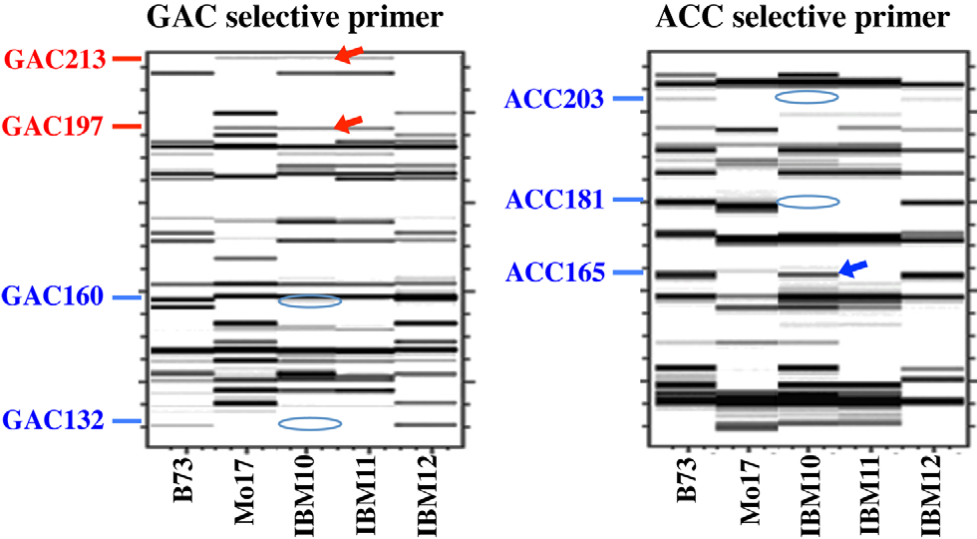
The B73_8_ACC165 gene conversion event. This figure illustrates marker gain as primary data; see also Figure S2 for a visualization of how the data are interpreted. Panels show gel images acquired using fluorescent (FAM) labeling and capillary electrophoresis (images produced by GeneMarker software). IBM10 contains all Mo17 markers from centromere 8 as well as the centromere 8 B73_8_ACC165 marker (B73 markers are labeled in blue and Mo17 markers are labeled in red). IBM11 and IBM12 contain normal Mo17 and B73 centromeres, respectively. Only a subset of the (total 30) markers for centromere 8 is shown; see Figure S2 for the complete list.

The IBM population presents a unique opportunity for identifying rare genetic exchanges within centromere cores. Since crossing over is suppressed in centromeres, the markers from a single centromere haplotype should always be inherited as a unit. While this is true for the great majority of centromeres, we also detected aberrant inheritance patterns. These fell into two categories: loss of a marker from a known centromere haplotype and gain or transfer of a marker from one haplotype to another (Figure 3). Marker loss is a negative result and difficult to confirm; such events may in principle represent deletions but could potentially represent technical errors and were thus not pursued further. In contrast, there are several definitive ways to confirm the gain of a marker in our scoring system, and we focused further analyses on these markers.

There were four cases of marker gain, each potentially representing a genetic exchange event. We first cloned and sequenced each affected band from its parental line. We then performed a new round of TD using sequence-specific primers. In two such cases, the originally scored gained bands were not observed using the sequence-specific primers, indicating that the bands likely represent new polymorphisms that happened to co-migrate with one of the mapped markers. Two other bands—B73_8_ACC165 and Mo17_5_TCG264—were confirmed by sequence to represent the parental markers. At least one of these markers (B73_8_ACC165) lies within the functional CENH3 core as assayed by ChIP display. The second marker (Mo17_5_TCG264) did not precipitate with CENH3 antisera in our hands, though we note that a negative result by ChIP does not necessarily imply that the marker is not centromeric.

An analysis of flanking markers revealed that no crossing over was associated with either B73_8_ACC165 or Mo17_5_TCG264, ruling out the possibility that they represent crossing over at the edge of the affected centromeres and indicating that they represent gene conversion, double crossover, or similar sequence exchange events (Figure S2). It is also possible (though much less likely) that these events represent exchange between non-homologous centromeres. Although we have not demonstrated that the observed marker exchanges are mechanistically gene conversion in the strictest sense, we will refer to them as conversion events throughout. Based on these observations, we can estimate that the IBM lines sustained a centromeric gene conversion rate of 1.86×10^−4^ conversion events per marker per generation (see Materials and Methods).

### Linkage Disequilibrium (LD) in Maize Centromeres

**Figure 4:**
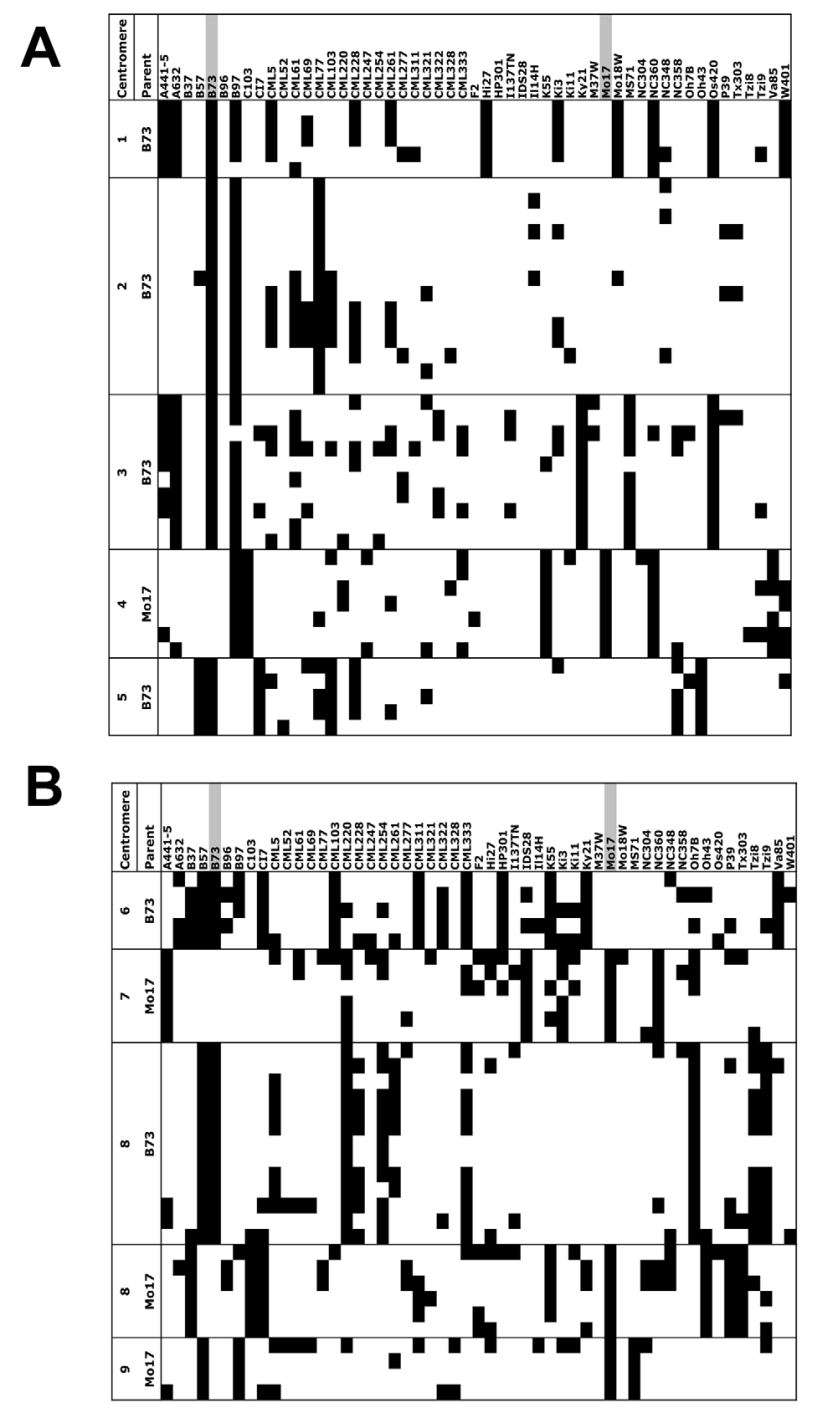
CRM2 marker data from a set of diverse inbreds. Panels A and B together represent the entire data set. Columns show the 53 inbreds scored, while rows show the presence (black) or absence (white) of 75 CRM2 TD markers for the indicated centromeres. The columns containing B73 and Mo17 reference data are highlighted in grey. For centromere 2, only sequence-confirmed data are shown, whereas all other data were interpreted with a presumed false positive rate of 1.8%.

Direct observation of marker exchange in our mapping population confirms the existence of conversion events, but population genetic data are required to assess the historical impact that such processes may have had on maize centromeres. To this end, we genotyped a set of CRM2 TD markers in a panel of 53 inbred lines, including a 50-line core set representative of a broad base of maize genetic diversity [34]. Each line was genotyped with 75 markers derived from 10 centromeres (B73 centromeres 1, 2, 3, 5, 6, 8, and Mo17 centromeres 4, 7, 8, and 9; Figure 4). When scoring CRM2 markers in diverse inbreds, there is a possibility that unrelated bands might co-migrate with the B73- or Mo17-derived bands and thus be scored as false positives. To investigate this possibility, we confirmed all bands for a set of 12 sequenced markers on centromere 2 [24] using a second round of genotyping using 4 bp selective base primers. The data revealed that 98.2% of the genotypes (556 of 566) from centromere 2 had been scored correctly. The remaining data are reported as originally called with 3 bp primers and interpreted with an assumed false positive rate of 1.8% (Figure 4).

Because all of the assayed lines are inbred, it is reasonable to interpret our multi-locus genotypes as haplotypes for population genetic analysis, even though the markers are genetically dominant. Initial investigation of average pairwise LD among markers, as measured by the Z_nS_ statistic [35], revealed that observed haplotype configurations at 7 of the 9 centromeres cannot be explained by a model lacking historic genetic exchange (Table 1). To further test for evidence of genetic exchange, we applied the four-gamete test [36] to estimate the minimum number of genetic exchanges (Rmin) required to explain the observed data (assuming no recurrent mutation). As shown in Table 1, all nine centromeres were estimated to have nonzero Rmin (mean = 5.6), providing strong evidence for some form of genetic exchange. These Rmin values, moreover, are likely underestimates of the actual number of exchanges that have occurred at each centromere, as our markers cover only a small region of each centromere and Rmin is an inherently conservative statistic [36].

**Table 1:** Linkage disequilibrium and gene conversion rates.

Centromere	Markers	Rmin	Z_nS_	*N*	Gene Conversion Rate^1^	
					Simulation	Likelihood
1	5	2	0.586	7	1.04	0.35
2	14	8	0.386**	13	1.04	0.91
3	10	8	0.326**	24	5.09	1.40
4	7	5	0.379**	14	4.09	1.42
5	5	4	0.487	10	0.461	0.90
6	5	4	0.320**	12	8.48	0.85
7	6	4	0.282**	12	8.18	1.36
8_B73	13	7	0.445*	19	2.12	0.36
8_Mo17	6	5	0.325**	14	3.64	0.91
8^2^	19	13	0.249**	33	3.64	0.90
9	4	2	0.312**	6	1.62	0.93

N = number of haplotypes.

1 Rates presented as conversions per 10^5^ markers.

2 All centromere 8 data combined.

*p<0.05, ** *p*<0.001.

**Figure 5:**
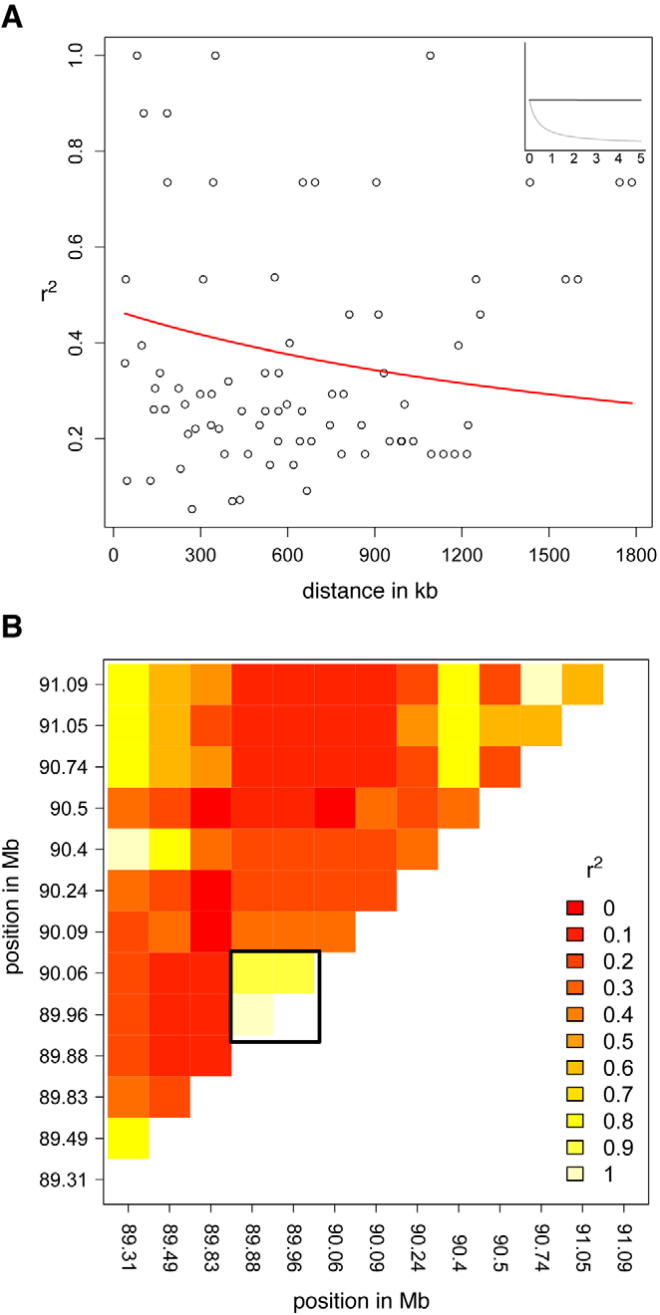
Linkage disequilibrium in centromere 2. (A) Pairwise LD plotted against distance, fit to a decay function [37],[49] using a value of ρ = 8.81×10^−7^. Inset shows the decay over the first 5 kb (in black) and the same function fit using the genome-wide median of ρ (in grey) [51]. (B) Heatmap of pairwise LD. Lighter colors show higher LD. The black box demarcates three markers that show high LD and flank the only cluster of CentC repeats on this centromere (the 180 kb region between positions 89.88 and 90.06 Mb on the physical map).

Genetic exchanges such as those measured by Rmin can be caused by either crossing over or gene conversion. These two types of exchange result in different predictions about the relationship between LD and physical distance. Crossing over produces a negative correlation between LD and distance. For instance, LD on maize chromosome arms decays to negligible levels within 2 kb [37]. In contrast, because gene conversion tracts are usually short [38] and do not affect flanking markers, gene conversion is not expected to produce a relationship between marker distance and linkage. We measured the relationship between LD and distance on centromere 2 (Figure 5), which has been fully sequenced [24]. Pairwise LD estimates reveal a block of high LD involving 3 markers spanning the only region of CentC repeats on this centromere ([24]; marked as a box on Figure 5B), but the data reveal no evidence for a correlation between LD and distance (Pearson's correlation coefficient of 0.11 does not differ from randomly permuted datasets; *p* = 0.32). This pattern differs dramatically from what has been observed in the rest of the genome (Figure 5, inset) [37]. Moreover, forcing the data to fit a model of nonlinear decay [37] results in an estimate of crossing over of 3.94×10^−12^ per bp per generation—so low as to be inconsequential. These results are thus inconsistent with the observed genetic exchange being the result of canonical crossing over.

**Figure 6:**
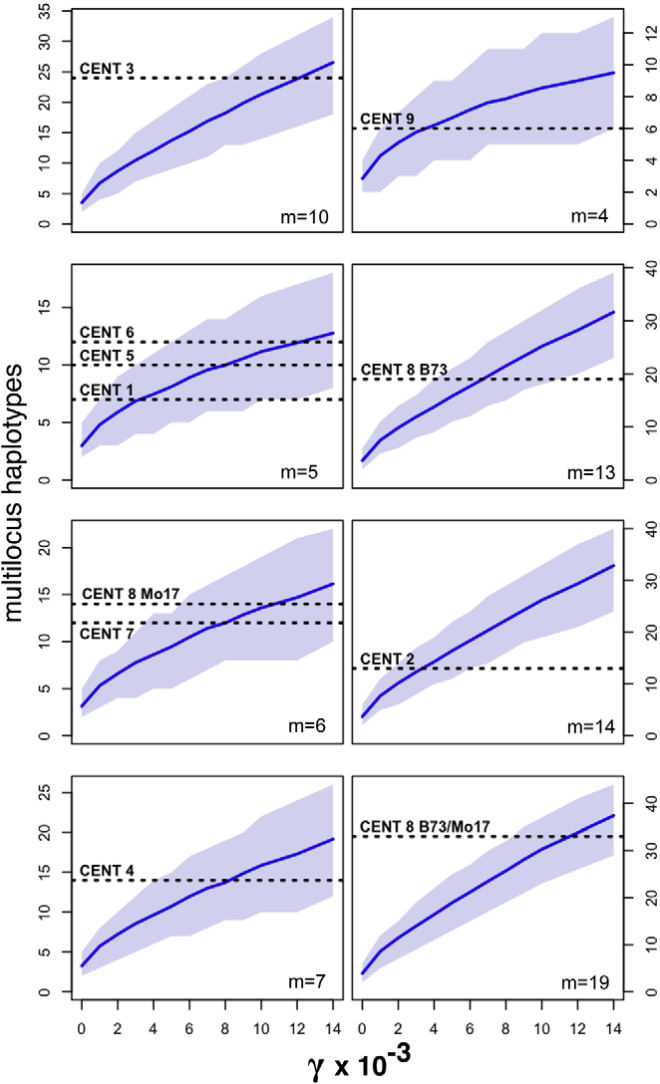
Haplotype estimation of gene conversion. Shown is the expected number of haplotypes observed under varying levels of gene conversion (γ) from coalescent simulations of a maize domestication bottleneck. The solid line indicates the mean number of haplotypes, and the shaded region encloses the empirical 95% confidence intervals. Horizontal dotted lines represent the number of haplotypes observed from the centromeres indicated (m is the number of markers in that centromere). The most probable gene conversion rates occur where the dotted lines intersect with the solid lines. The last panel shows the outcome if all centromere 8 data are considered together (from both B73 and Mo17, such that m = 19).

We therefore proceeded to estimate the rate of gene conversion on each centromere using two independent methods (Table 1). The first is based on the premise that gene conversion will increase the number of multilocus haplotypes in a sample. Coalescent simulations (see Materials and Methods; Figure 6) were used to estimate the gene conversion rate required to achieve the observed number of haplotypes. The resulting data suggest a mean estimate of 3.7×10^−5^ conversion events per marker per generation and allow us to statistically reject a model with no gene conversion for all nine centromeres at *p*<0.05. Second, we used a composite likelihood method [39] to directly estimate gene conversion rates for each centromere. This second approach reveals similar rates of conversion across all nine centromeres, averaging ∼1×10^−5^ conversion events per marker per generation.

## Discussion

Our data indicate that gene conversion is common within centromeres and may play a fundamental role in determining the dynamics and distribution of centromere repeats. This conclusion is based on three primary lines of evidence. First, our mapping data provide what is to our knowledge the only experimental evidence for centromeric gene conversion. Indeed, two independent conversion events were identified in 93 recombinant inbred lines using a set of 238 CRM2 markers, corresponding to a rate of 1.86×10^−4^ exchanges per marker per generation. The second line of evidence comes from LD analysis of 75 markers typed in a set of 53 diverse inbred lines. These data show patterns consistent with genetic exchange, including unusually low LD and the clear presence of recombinant haplotypes (nonzero Rmin), but show no decay of LD with distance as would be expected in the presence of crossing over. Finally, two independent population genetic methods were used to directly estimate centromeric gene conversion, resulting in remarkably similar rates of ∼1×10^−5^ conversions per marker per generation. It is too early to tell how rates of gene conversion in centromeres compare to other regions of the maize genome, but one estimate of gene conversion at the maize *anthocyaninless1* locus (∼3×10^−5^/marker/generation [40]) suggests they may be of a similar order of magnitude.

It has been hypothesized that centromere evolution in eukaryotes with asymmetric meiosis has been primarily governed by an arms race in which meiotic drive occasionally sweeps novel centromeric repeats to fixation [4]. While the extreme LD observed around a short tract of CentC on centromere 2 may hint at an evolutionary history consistent with these ideas (Figure 5B), our finding of widespread gene conversion explains how high levels of diversity may be observed even in yeast where meiotic drive is a less likely explanation [7]. Sequence data from mammalian centromeres are further consistent with this view, suggesting in several studies that gene conversion has contributed to extant centromere variation and the production of novel higher order repeat arrays [17],[18],[19]. If centromeric gene conversion is indeed common in maize, yeast, and humans, it seems reasonable to hypothesize that gene conversion is an important process within the centromere cores of all eukaryotes.

## Materials and Methods

### Genetic Stocks

A ninety-four line IBM DNA Kit, provided by the Maize Genetics Cooperation Stock Center (http://www.maizemap.org/94_ibm.htm), was used for CRM2 display. IBM3 was excluded from the analysis because seven centromeres were heterozygous. Additional accessions of IBM lines used for confirmation and further ChIP and FISH analysis were obtained from the Maize Genetics COOP stock center (http://www.maizegdb.org/stock.php).

A set of 53 maize inbred lines, including the majority of a 50-line core set [34] with additional lines within NAM (nested association mapping) founder lines [41], were chosen to represent the genetic diversity for LD analysis. The inbreds assayed were B73, Mo17, A441, A632, B37, B57, B96, B97, C103, CI.7, CML5, CML52, CML61, CML69, CML77, CML103, CML220, CML228, CML247, CML254, CML261, CML277, CML311, CML321, CML322, CML328, CML333, F2, Hi27, HP301, I137TN, IDS28, IL14H, K55, Ki3, Ki11, KY21, M37w, Mo18w, Ms71, Nc304, Nc360, Nc348, Nc358, Oh7B, Oh43, Os420, P39, Tx303, Tzi8, Tzi9, Va85, and W401. All were obtained from the North Central Regional Plant Introduction Station, in Ames, Iowa. DNA was extracted from 3-wk-old seedlings using a modified CTAB protocol [42].

### CRM2 Transposon Display

Transposon display was carried out as described elsewhere [24],[29]. In this method, DNA is digested with *Bfa*I and the samples PCR-amplified using CRM2 primers and adapter primers designed to anneal to the cleaved *Bfa*I site. The method involves primary and selective amplification steps with different (nested) CRM2 primers being used in each step. The primers for primary amplification were CRM2_R1 (5′- GAGGTGGTGTATCGGTTGCT) and *Bfa*I + 0 (5′- GACGATGAGTCCTGAGTAG), and for selective amplification were P^33^ or FAM-labeled CRM2_R2 (5′-CTACAGCCTTCCAAAGACGC) and *Bfa*I + 3 selective bases (where different bases were added to the Bfa + 0 primer). A 58°C annealing temperature was used for the selective amplification. P^33^-labeled PCR products were separated on 6% polyacrylamide gels and FAM-labeled PCR products were separated by capillary electrophoresis and interpreted using GeneMarker software (SoftGenetics, LLC).

### Genetically Mapping CRM2 Markers

Mapping data were initially sent to a community IBM mapping service (CIMDE), which constructed a linkage map using a two-point mapping method from a framework of 580 loci. After obtaining rough positions, we constructed a finer centromere map for each chromosome using MapMaker Version 3.0 [43]. In each centromere map, mapping scores for 20 flanking markers from the IBM2 2008 Neighbors linkage interpretation (www.maizegdb.org) were added to the file containing CRM2 markers scores. The closest IBM2 core bin markers were added as the first and last marker for each centromere map. In addition, we included as many “skeleton” markers (ISU map4, [13]) as possible. The CRM2 markers were then placed into the centromere framework using a multi-point method (the “try” MapMaker command).

### Identifying CENH3-Associated Markers by ChIP Display

Native ChIP was carried out as described previously [44] with minor modifications. Chromatin was extracted from young leaves (∼8–15 cm) or young roots (∼1 wk after germination). RNase-free DNase I (Promega, Madison, WI, USA) was utilized for chromatin digestion. Chromatin was digested to ∼300–3,000 bp fragments as judged by agarose electrophoresis. After immunoprecipitation with anti-CENH3 antisera [23], the supernatant (unbound) and IP (bound) fractions were purified with a PCR purification kit (Invitrogen, Carlsbad, CA, USA) and used for CRM2 transposon display. Input DNA (before adding antibodies) was used as a positive control and a treatment without antibodies (No IgG) was used as a negative control (Figure 2). ChIP display was replicated three times for both B73 and Mo17; bands that were amplified in the IPed DNAs from all three experiments were considered to be associated with centromere cores.

### Recovery and Sequencing of CRM2 Markers

Sixty-four CRM2 bands were excised from TD gels and re-amplified with primer set BfaI+0 and CRM2_R2. The PCR products were purified using QIAGEN (Valencia, CA) Gel Purification kit and were either directly sequenced or cloned into a TOPO TA vector (Invitrogen, Carlsbad, CA) and then sequenced. As controls for the ChIP display method, 31 bands were cloned from both genomic DNA and ChIP display (IP) lanes, and the resulting sequences were found to be identical.

All sequenced markers are available in GenBank as accessions GF099546–GF099610. Markers that were shown to interact with CENH3 are annotated with the statement “this sequence interacts with Centromeric Histone H3 (CENH3) and is within the functional centromere core.” We note that a subset of the sequenced markers was also used to construct the physical map of centromeres 2 and 5 [24].

### Identifying and Confirming Heterozygous Centromeres in IBM Lines

Heterozygous centromeres were first identified as cases where markers from both parents were present for a single centromere. A total of 27 such examples were identified. Seven heterozygous centromeres were found in a single line (IBM3) that was subsequently removed as a recent outcross contaminant. We made an effort to confirm as many of the remaining 20 heterozygous centromeres as possible using codominant insertion-deletion polymorphisms (IDPs; [13]) to confirm heterozygosity at closely linked flanking markers (16 centromeres) or by FISH of CentC content (one centromere, Figure S1). We were also able to eliminate as contaminants six centromeres that lacked markers from either parent and were together responsible for all of the non-parental bands observed on TD gels. Although they lacked B73 or Mo17 markers, four of the contaminant centromeres were shown to contain abundant CentC and CRM and one line segregated for knobs not present in either parent (Figure S1).

The IDPs scored were IDP3936, IDP592, and IDP825 (chromosome 2); IDP3945 and IDP1433 (chromosome 3); IDP642, IDP476, and IDP625 (chromosome 4); IDP1408, IDP359, and IDP1607 (chromosome 5); IDP3788, IDP3799, IDP2581, IDP680, and IDP3887 (chromosome 6); IDP3795, IDP3810, and IDP3994 (chromosome 7); IDP334, IDP327, IDP811, and IDP88 (chromosome 8); and IDP4151, IDP8457, and IDP4017 (chromosome 9).

### Confirming Gene Conversion Events

Two gene conversion events identified by B73_8_ACC165 and Mo17_5_TCG264 were confirmed in several experiments using different DNA samples and primers. The most definitive experiment for marker B73_8_ACC165 involved a highly specific primer with 11 selective bp. With this primer, the segregation was identical to the original observation, such that RIL IBM10, which contains the complete Mo17 centromere 8 haplotype, also contains marker B73_8_ACC165 from B73 centromere 8. For marker Mo17_5_TCG264, we directly sequenced the aberrantly scored bands in the affected RILs IBM24 and IBM54. Both lines contain the complete B73 centromere 5 haplotype as well as the Mo17_5_TCG264 marker from Mo17 centromere 5.

We ruled out that crossover had occurred coincidently with marker gain using our established centromere map positions [24]. For centromere 5 we used the following markers: umc40, mmp60, rz87 - Cent5 - umc1591, umc2302, and umc1060. For centromere 8 we used bnlg1834, umc1157, umc1904 - Cent8 - AY110113, gpm572b, and IDP334. Map scores for the flanking gene markers have been previously published [13],[45] and were obtained from maizegdb.org.

### FISH

FISH on mitotic cells was performed as described previously [32]. The following four repetitive DNA sequences were included in the probe cocktail: subtelomeric 4-12-1 (FITC labeled), CRM2 LTR (FITC labeled), CentC (Texas Red labeled), and knob180 (Texas Red labeled). The clones of 4-12-1, CentC, and knob180 were generously provided by Dr. James Birchler (University of Missouri). The CRM2 LTR was PCR amplified from genomic DNA using the following primer set: forward, 5′-TCGTCAACTCAACCATCAGGT, and reverse, 5′-GCAAGTAGCGAGAGCTAAACTTGA. All images were captured and processed using a Zeiss Axio Imager microscope and SlideBook 4.0 software (Intelligent Imaging Innovations, Denver, CO, USA).

### Estimation of Gene Conversion Rate in IBM Lines

Assuming that all markers have equal likelihood of being involved in an exchange event, and taking into account the decrease in heterozygosity during the 11 generations involved in preparing the mapping population, we can estimate the rate of gene exchange as 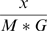, where *x* is the observed number of exchanges, *M* the total number of markers, and *G* the effective number of generations available for exchange. We observed two exchange events, and scored 238 markers in each of the 93 lines remaining after removing contamination. A further 696 markers were removed because of contamination or inconsistent banding patterns, such that the total number of markers was *M* = 21,438. In a randomly mating population, all 11 generations would provide opportunities for exchange. But as RILs are inbred, each generation possesses less heterozygosity and thus fewer opportunities to observe an exchange event. Correcting for this, the effective number of generations is 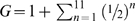, and the total rate is 1.86×10^−4^ exchanges per marker per generation.

### LD and Simulation

Calculation of Rmin, pairwise r^2^, and Z_nS_ utilized code from the analysis and msstats packages of the libsequence C++ library [46]. We modeled the decay of LD with distance [37] and tested the significance of the association between r^∧^2 and distance along centromere 2 with 1,000 pairwise permutations. The significance of the Z_nS_ statistic for each centromere was compared to results from 1,000 coalescent simulations under a bottleneck model (similar to [47]) with no recombination. Simulations were performed in ms [48] with the command line:

ms 53 1000 -t 500 -r 0 1000000 -c γ 1000 -eN 0.00556 0.00544 -eN 0.00611 1.

### Estimation of Gene Conversion in Diverse Inbreds

We used two independent methods to estimate gene conversion rates. First, composite likelihood methods [39], as implemented in the program maxhap (http://home.uchicago.edu/~rhudson1/source/maxhap.html), were used to estimate the population gene conversion rate γ ( = 4N_e_g), where g is the gene conversion rate per bp per generation. We assumed a gene conversion tract length of 1 kb, a population recombination rate of ρ = 4N_e_r = 10^−5^ per kb, where r is the recombination rate per bp per generation, and that markers were evenly spaced across the centromere. Centromere sizes were based on map estimates [24]. Physical map positions from centromere 2 were utilized to verify that assumptions of order and distance had little effect on the final rate estimation (unpublished data). Using maxhap, we calculated the likelihood of different rates across a grid of 10,000 values of γ/ρ from 1 to 10^6^ per kb, reporting the value of γ which maximized the likelihood for each centromere.

Our second estimator of gene conversion compared the number of multilocus haplotypes present in a sample of centromere markers to coalescent simulations under a demographic model of maize domestication. We simulated chromosomes nearly devoid of recombination across a grid of gene conversion rates, performing 1,000 coalescent simulations for each value investigated. Our model closely followed prior work [47] in assuming an ancestral diploid population size of 450,000 that underwent a domestication bottleneck of 2,450 individuals, starting 11,000 years ago and lasting 1000 years. Simulations were performed in MaCS [50] using the following command line:

macs 53 10e6 -t 10e-3 -r 10e-6 -c γ 1000 -eN 0.00556 0.00544 -eN 0.00611 1-h 10e5.

Custom programs built using the libsequence C++ library [46] were used to ascertain markers using a scheme mirroring our TD methods, to choose a random subset of markers for comparison to different centromeres, to incorporate a false positive error rate of 1.8% (i.e., randomly change marker absence to marker presence with a probability of 1.8%), and to count haplotypes from the resulting simulated data.

In both cases, to extract the rate g from our estimates of γ, we calculated the effective population size N_e_ from the mean genome-wide nucleotide diversity in maize [51] assuming a mutation rate of 3×10^−8^ [52]. To calculate conversion rates on a per marker basis, we assumed the average tract length to be 1 kb and the average CRM2 marker to be 200 bp long.

## Supporting Information

Figure S1 **Confirmation of centromere heterozygosity and contamination by FISH.** (A) A chromosome spread from IBM85, showing centromere heterozygosity at chromosome 4. Note the differing amount of red (CentC) signal on the circled chromosomes. (B) A gel image showing that IBM47 and IBM85 are heterozygous in centromere 4 flanking regions. These data show the results for the IDP476 marker. Molecular weights of the size standards (in bp) are also indicated. (C) A chromosome spread from a cross between IBM58 and B73, showing a chromosomal feature (a knob, in red) on chromosome 2 that is not present in either B73 or Mo17. CentC (faint) and the knob 180 bp repeat are shown in red, CRM2 LTR and telomeres are shown in green, and chromosomes are shown in blue.
(1.66 MB TIF)

Figure S2 **A complete list of markers from centromere 8 covering the** ***bnlg1834*** **to** ***IDP334*** **interval and the genotypes of IBM10, 11, and 12.** Map scores for the six flanking gene markers have been previously published [13],[45] and were obtained from maizegdb.org. The distances in centromere-flanking regions are shown in IBM cM units, which equate to roughly one fourth the size of a standard cM. The seven Mo17 within-centromere markers and 23 B73 within-centromere markers are distributed randomly and are not meant to convey actual distance or order relative to each other (all 30 markers map genetically to the same location). For each of the IBM genotypes, B73 polymorphisms are represented by the letter B and Mo17 polymorphisms are represented by the letter M.
(0.55 MB TIF)

Table S1 **Heterozygosity, contamination, and gene conversion in IBM lines.** ^1^ het = heterozygous; / = contaminant centromere; gc = gene conversion. ^2^ IBM3 was removed.
(0.23 MB DOC)

## Abbreviations

CENH3: Centromeric Histone H3
ChIP: chromatin immunoprecipitation
FISH: fluorescent in situ hybridization
IDPs: insertion-deletion polymorphisms
LD: linkage disequilibrium
