# Supplementary figures and images for: Widespread Gene Conversion in Centromere Cores
Source: PLoS Biol. 2010 Mar 9;8(3):e1000327. doi: 10.1371/journal.pbio.1000327 (PMC2834711; doi:10.1371/journal.pbio.1000327)

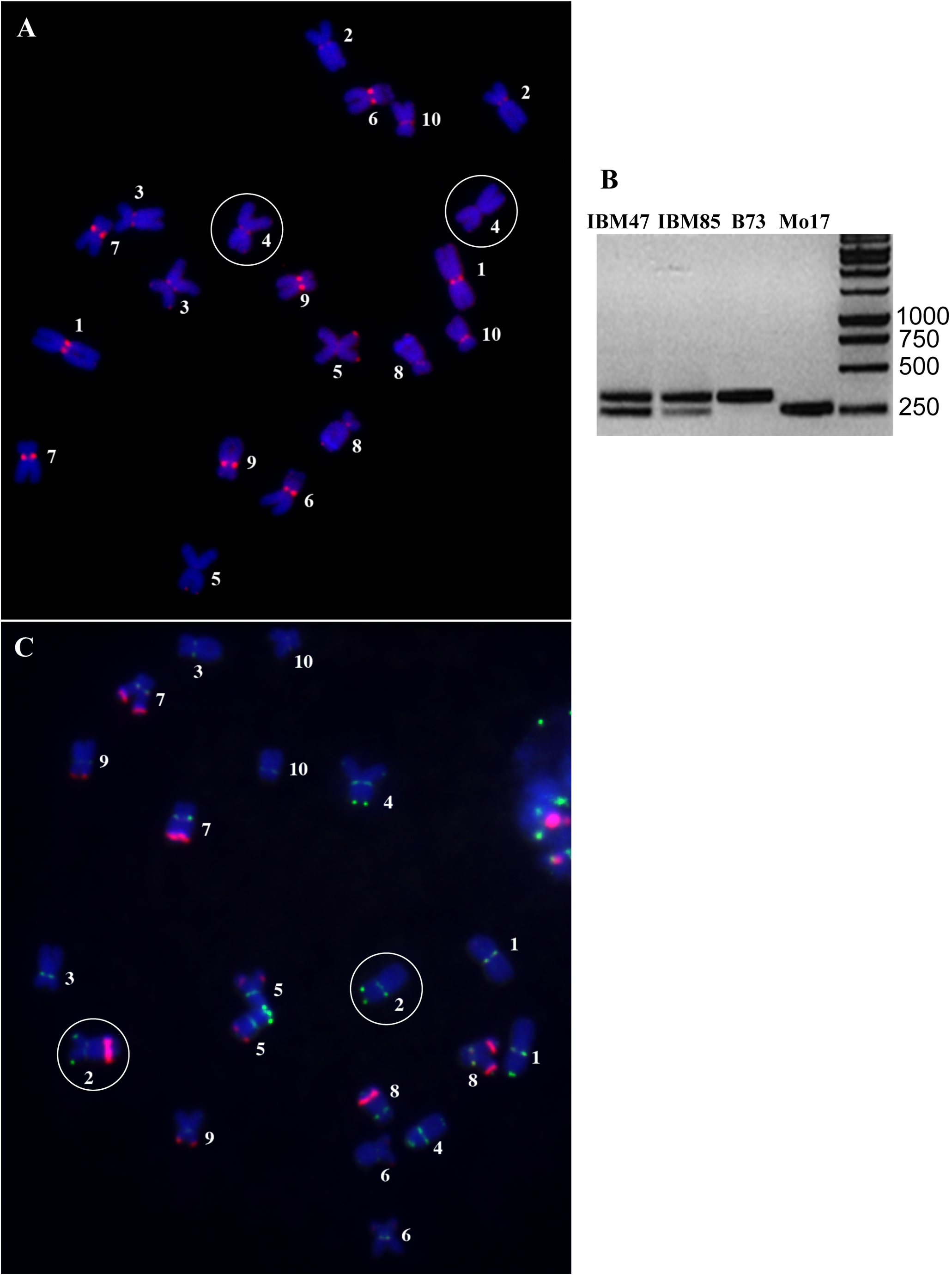

Supplement: Figure S1 — Confirmation of centromere heterozygosity and contamination by FISH. (A) A chromosome spread from IBM85, showing centromere heterozygosity at chromosome 4. Note the differing amount of red (CentC) signal on the circled chromosomes. (B) A gel image showing that IBM47 and IBM85 are heterozygous in centromere 4 flanking regions. These data show the results for the IDP476 marker. Molecular weights of the size standards (in bp) are also indicated. (C) A chromosome spread from a cross between IBM58 and B73, showing a chromosomal feature (a knob, in red) on chromosome 2 that is not present in either B73 or Mo17. CentC (faint) and the knob 180 bp repeat are shown in red, CRM2 LTR and telomeres are shown in green, and chromosomes are shown in blue. (1.66 MB TIF) [file pbio.1000327.s001.tif]

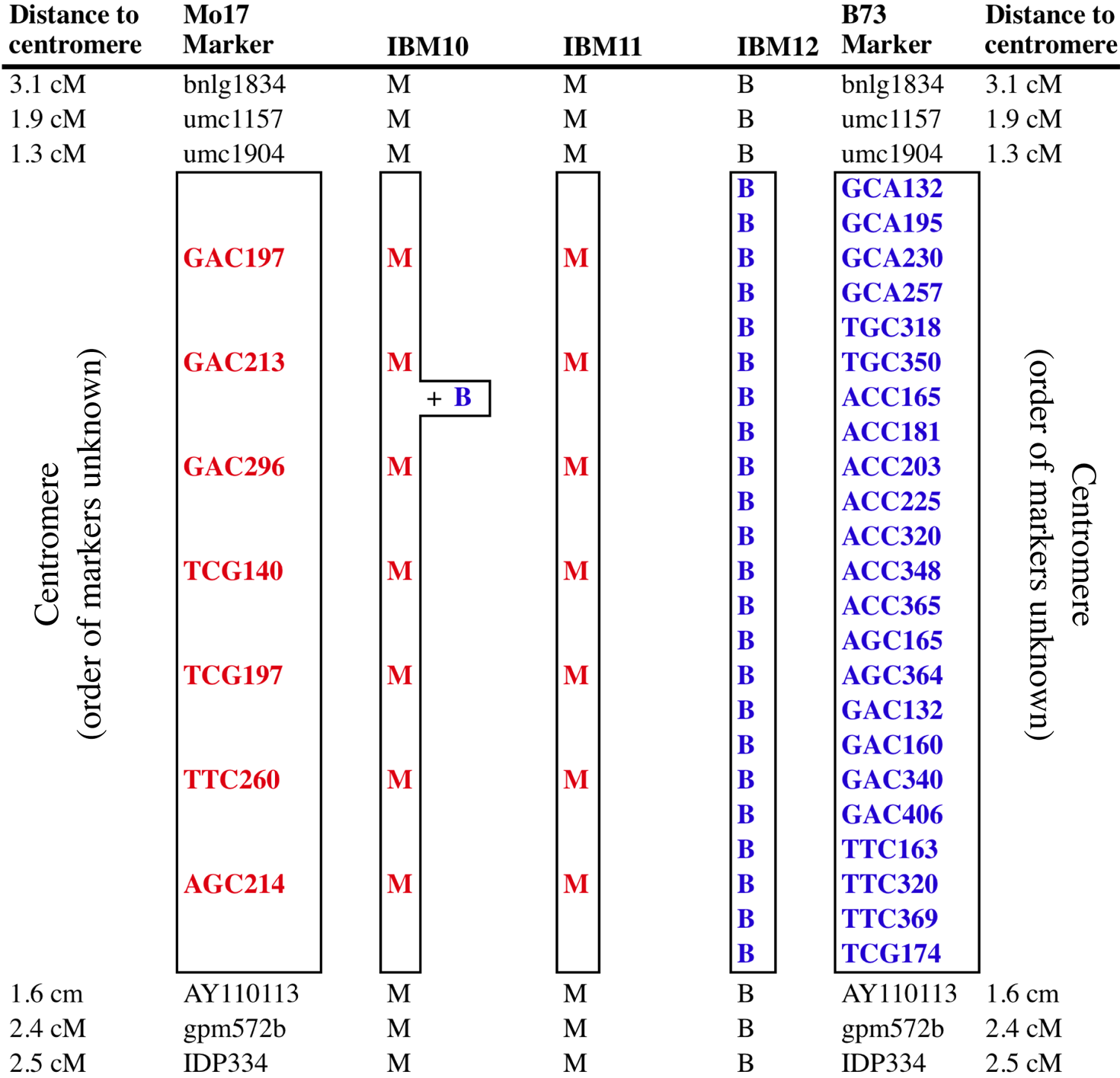

Supplement: Figure S2 — A complete list of markers from centromere 8 covering the bnlg1834 to IDP334 interval and the genotypes of IBM10, 11, and 12. Map scores for the six flanking gene markers have been previously published [13],[45] and were obtained from maizegdb.org. The distances in centromere-flanking regions are shown in IBM cM units, which equate to roughly one fourth the size of a standard cM. The seven Mo17 within-centromere markers and 23 B73 within-centromere markers are distributed randomly and are not meant to convey actual distance or order relative to each other (all 30 markers map genetically to the same location). For each of the IBM genotypes, B73 polymorphisms are represented by the letter B and Mo17 polymorphisms are represented by the letter M. (0.55 MB TIF) [file pbio.1000327.s002.tif]

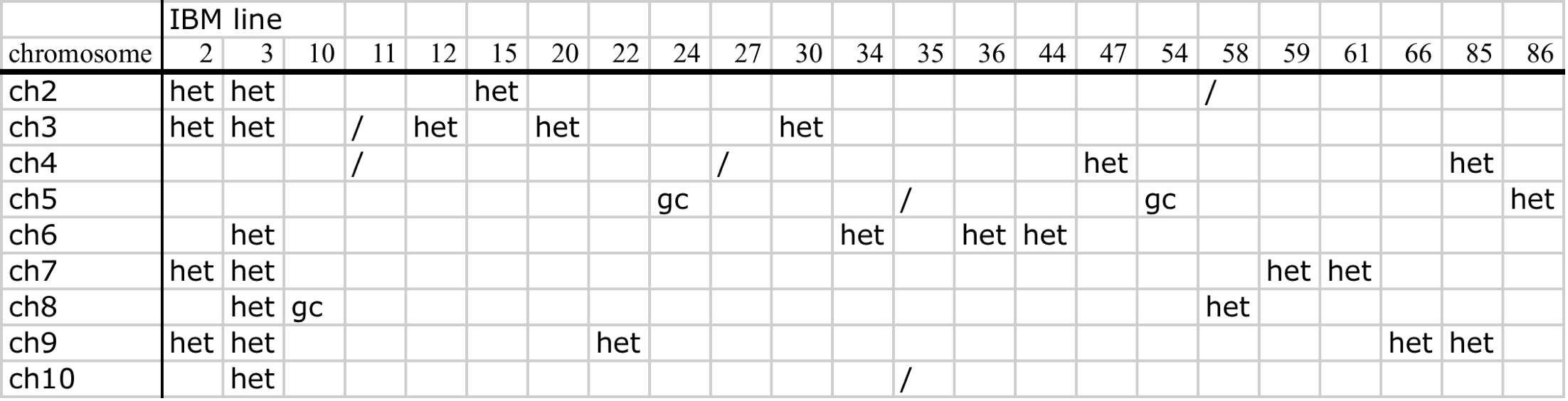

Supplement: Table S1 — Heterozygosity, contamination, and gene conversion in IBM lines. 1 het = heterozygous; / = contaminant centromere; gc = gene conversion. 2 IBM3 was removed. (0.23 MB DOC) [file pbio.1000327.s003.doc]
